# Effects of BEIIb-Deficiency on the Cluster Structure of Amylopectin and the Internal Structure of Starch Granules in Endosperm and Culm of *Japonica*-Type Rice

**DOI:** 10.3389/fpls.2020.571346

**Published:** 2020-11-17

**Authors:** Yasunori Nakamura, Masami Ono, Tamao Hatta, Keiji Kainuma, Kazuki Yashiro, Go Matsuba, Akira Matsubara, Akio Miyazato, Goro Mizutani

**Affiliations:** ^1^Starch Technologies, Co., Ltd., Akita Prefectural University, Akita, Japan; ^2^Akita Natural Science Laboratory, Katagami, Japan; ^3^Faculty of Risk and Crisis Management, Chiba Institute of Science, Choshi, Japan; ^4^Science Academy of Tsukuba, Tsukuba, Japan; ^5^Graduate School of Organic Materials Science, Yamagata University, Yonezawa, Japan; ^6^School of Materials Science, Japan Advanced Institute of Science and Technology, Nomi, Japan

**Keywords:** cereal endosperm, amylopectin structure, small angle X-ray scattering, starch branching enzyme IIb, starch biosynthesis

## Abstract

It is known that one of starch branching enzyme (BE) isoforms, BEIIb, plays a specific role not only in the synthesis of distinct amylopectin cluster structure, but also in the formation of the internal structure of starch granules in rice endosperm because in its absence the starch crystalline polymorph changes to the B-type from the typical A-type found in the wild-type (WT) cereal endosperm starch granules. In the present study, to examine the contribution of BEIIb to the amylopectin cluster structure, the chain-length distributions of amylopectin and its phosphorylase-limit dextrins (Φ-LD) from endosperm and culm of a null *be2b* mutant called *amylose-extender* (*ae*) mutant line, EM10, were compared with those of its WT cultivar, Kinmaze, of *japonica* rice. The results strongly suggest that BEIIb specifically formed new short chains whose branch points were localized in the basal part of the crystalline lamellae and presumably in the intermediate between the crystalline and amorphous lamellae of amylopectin clusters in the WT endosperm, whereas in its absence branch points which were mainly formed by BEI were only located in the amorphous lamellae of amylopectin. These differences in the cluster structure of amylopectin between Kinmaze and EM10 endosperm were considered to be responsible for the differences in the A-type and B-type crystalline structures of starch granules between Kinmaze and EM10, respectively. The changes in internal structure of starch granules caused by BEIIb were analyzed by wide angle X-ray diffraction, small-angle X-ray scattering, solid state ^13^C NMR, and optical sum frequency generation spectroscopy. It was noted that the size the amylopectin cluster in *ae* endosperm (approximately 8.24 nm) was significantly smaller than that in WT endosperm (approximately 8.81 nm). Based on the present results, we proposed a model for the cluster structure of amylopectin in WT and *ae* mutant of rice endosperm. We also hypothesized the role of BEIIa in amylopectin biosynthesis in culm where BEIIb was not expressed and instead BEIIa was the major BE component in WT of rice.

## Introduction

Starch is composed of an essentially linear or scarcely branched glucan called amylose and a highly branched glucan called amylopectin, both components accounting for 15–30% and 70–85%, respectively. In plant tissues native starch occurs in the form of granules, which vary in sizes (0.1–200 μm in diameter) and shapes (spheres, ovals, ellipsoids, polygons, platelets, disks, and rods) depending on plant species and tissues (Kerr, 1950; Sterling, 1968; Jane, 2009). The fine structure of amylopectin greatly differs from that of glycogen in several ways. Amylopectin has a unit structure designated as cluster whereas glycogen has no such unit structure because *upalpha*-1,6 branch points are randomly distributed in glycogen (Thompson, 2000). The clusters of amylopectin are interconnected by long chains designated as B2 chains and/or B3 chains that span to two clusters and/or three clusters, respectively, whereas short and intermediate chains within a single cluster are called A chains (non-branched chains) or B1 chains (branched by at least one chains) (Peat et al., 1952). Thus, after the treatment of debranching with isoamylase the chain-length distribution of amylopectin forms a bimodal pattern whereas that of glycogen shows only a single peak in addition to the longer average chain-length in amylopectin compared with glycogen.

Nikuni (1969) had been questioning on the big difference of molecular weight of amylopectin determined by physicochemical methods and the Meyer’s tree like structure (Meyer and Bernfeld, 1940). He built a model for cluster using metal chains to produce a cluster of 900 glucose units composed of 30 linear glucose chains of DP30 connected at near the one reducing end. These cluster units could link together to form a gigantic molecular weight amylopectin in a starch granule. He also proposed the possibility of the cluster elongation to three dimensional directions and extremely assumed that all the clusters could connect to one C-chain. Independently, French (1972) proposed a cluster model based on the structure of branched malto-oligosaccharides (MOS) obtained by α-amylolysis of amylopectin. Roughly 65% of branch points were present as singly branched oligosaccharides, and 35% as doubly branched or triply branched MOS (Kainuma and French, 1970). Compared to the Meyer model, the French model proposed that the less-branched linear region of amylopectin reflects strongly in the X-ray diffraction pattern. The cluster models of amylopectin have been further developed by many research groups and researchers (Robin et al., 1975; French, 1984; Hizukuri, 1986; Bertoft, 2015), although the exact nature of its basic structure is still a matter of debate mainly because it is impossible to determine directly the positions of *upalpha*-1,6 branches of α-glucans, mainly due to limitation of analytical techniques (Bertoft, 2015).

One of the most striking features of the fine structure of amylopectin is the fact that the neighboring two chains form double helices in the region where the two chains are not branched and this region exceeds 10 glucosyl units or degree of polymerization (DP) of 10 in a parallel fashion extending their non-reducing ends outward (Gidley and Bulpin, 1987). The presence of double helices profoundly affects distinct physicochemical properties of starch granules. The formation of left-handed double helices between amylopectin side chains was first proposed by Kainuma and French (1972) based on the fact that native undissolved amylodextrin was stained only light yellow-brown color with iodine, in addition to the observation of X-ray diffraction analysis and the construction of space filling models. Details of the packing of the left handled double helix barrel in amylopectin molecules were later verified elaborately by Imberty and Pérez (1988) and Imberty et al. (1988, 1991). The cluster structure of amylopectin gives rise to the distinct crystalline nature of starch granules in which amylopectin molecules are regularly arranged by packing of parallel-stranded double helices and the lateral alignment of neighboring double helices (Yamaguchi et al., 1979; Kainuma, 1980; French, 1984). It has been reported that starch granules in cereal endosperm show the A-type crystalline polymorph whereas some tubers and rhizomes give the B-type crystalline polymorph, which have been generally distinguished by X-ray diffraction analysis (see review by Buléon et al., 1998). Although legume starches yield the C-type polymorph, which is revealed to be a mixture of A-type and B-type polymorphs (Bogracheva et al., 1998).

Past biochemical and genetic investigations have established that amylopectin and amylose can essentially be synthesized by three classes of the enzymes, namely, starch synthase (SS), starch branching enzymes (BE) and starch debranching enzymes (DBE) whereas each class of the enzyme has multiple isozymes having different enzymatic properties from each other (Nakamura, 2002, 2015). Although starch granules in the wild-type (WT) cereal endosperm exhibit the A-type polymorph, it is known that the *be2b*-mutations induce their A-type starches to the B-type starches in endosperms of maize (Gérard et al., 2000) and rice (Nishi et al., 2001; Tanaka et al., 2004), whereas no such changes have been so far reported in the *be1*- and *be2a*-mutations as well as *ss*-mutations (Satoh et al., 2003; Nakamura, 2018). The results indicate that BEIIb plays a specific role in the formation of fine structure of amylopectin and the starch crystalline structure in these endosperms.

The specific roles of BEIIb in the starch biosynthesis in rice endosperm have been extensively investigated. Analysis of chain-length distribution of amylopectin of the BEIIb-deficient mutant called *amylose-extender* (*ae*) indicates that BEIIb plays a specific role in the synthesis of outermost short chains of the amylopectin cluster because this role cannot be supplemented by BEIIa and BEI (Nishi et al., 2001; Tanaka et al., 2004; Nakamura et al., 2010). Thus, the *ae* mutant amylopectin is considered to have side chains with longer non-branched region of chains from non-reducing ends and the lower number of chains per single cluster compared with WT amylopectin (Nakamura, 2002, 2015). These changes in the fine structure of amylopectin cause the *ae* starch physicochemical properties to be resistant to thermal gelatinization and enzymatic hydrolysis (Nishi et al., 2001; Wei et al., 2010b; Tsuiki et al., 2016). The observations that the extents of the chain-length distribution and starch physicochemical properties are reported to be proportional to the BEIIb activity (Tanaka et al., 2004; Wang et al., 2018) suggest that BEIIb greatly affects not only the amylopectin fine structure but also the internal starch granule structure.

The relationship between the fine structure of amylopectin and the internal starch granule structure has not fully understood. In the present study, a *be2b* mutant line EM10 which was generated from a *japonica*-type rice cultivar Kinmaze was used as the experimental material because this *ae* mutant is known to have the B-type starch and its amylopectin has a markedly modified fine structure (Nishi et al., 2001). We aimed to clarify the contribution of BEIIb to the amylopectin fine structure and the starch granule crystalline structures. The structural features of the *ae*-amylopectin were analyzed and compared with those of its WT Kinmaze in details. We also compared the chain-length profile of amylopectin in the endosperms with that in the culms, which are known to function as the temporary reserve organ particularly slightly before the anthesis (Horie et al., 2005), but lack in BEIIb activity. It is known that the internal starch granule structures are composed of the complex hierarchical structure including the double helix, the A-type and B-type allomorph, the super-helix, the starch granule morphology, and the starch granule packing in plastids, ranging in size from 10^–4^ to 10^2^ μm (see reviews by Pérez and Bertoft, 2010; Blazek and Gilbert, 2011). Several physicochemical and microscopic methods have been used to analyze these structures, although every methodology has advantages and disadvantages. In this study, the internal starch granule structures were examined using both wide angle X-ray diffraction (XRD), small angle X-ray scattering (SAXS) (Yuryev et al., 2004), solid-state ^13^C NMR (Gidley and Bociek, 1985; Flanagan et al., 2013), and sum frequency generation spectroscopy (SFG) (Miyauchi et al., 2006; Li et al., 2012; Kong et al., 2014), because combined results obtained from all these methods might bridge the gap of our understanding of the features of ultra-structures of starch granules in rice endosperm at scales ranging from 1 nm to 10 μm (Blazek and Gilbert, 2011).

## Materials and Methods

### Reagents

Isoamylase from *Pseudomonas amyloderamosa* (PaISA) was provided by Hayashibara Co., Ltd. (Okayama, Japan). Phosphorylase a from rabbit muscle was obtained from SIGMA. AG 501-X8 (D) Resin (20–50 mesh) was purchased from Bio-Rad. The fluorophore 8-amino-1,3,6-pyrenesulfonic acid (APTS) was obtained from AB SCINEX (Tokyo, Japan). Glucose-1-phosphate was obtained from Wako Pure Chemical Industries, Ltd. (Tokyo). Percoll was obtained from MP Biomedicals (LLC., Illkirch, France).

### Plant Materials

A *BEIIb*-deficient mutant line EM10 and its allelic mutants were produced by treating fertilized egg cells of the rice WT *japonica*-type cultivar Kinmaze, as described previously (Nishi et al., 2001). Kinmaze and be2b mutants were grown at an experimental field of Akita Prefectural University under natural conditions. For analysis of amylopectin chain length distribution and physicochemical properties of starch granules, starch samples were prepared from mature kernels harvested in 2017. Culms were harvested just before the anthesis in 2019. Developing seeds were harvested about 15–20 days after pollination (DAP) in 2019. These samples were stored at −80°C before use.

### Preparation of Enzyme Extracts From Rice Endosperm and Culm

Five developing rice kernels at 15–20 DAP randomly selected from developing kernels harvested and pooled from about 10 individual plants were homogenized by hand by using a plastic pestle in a plastic tube on ice with 250 μl of a grinding solution (GS) consisting of 50 mM imidazole-HCl (pH 7.4), 8 mM MgCl_2_, 5 mM dithiothreitol, and 12.5% (v/v) glycerol. The homogenate was centrifuged at 10,000 *g* for 20 min at 4°C, and the supernatant was again centrifuged at the same condition. The resulting supernatant was referred to as the crude enzyme extract and used for zymogram analysis of activities of BE isozymes, namely BEI, BEIIa, and BEIIb.

About 1 g (fresh weight) of the culm randomly selected from culms harvested and pooled from about 5 individual plants was cooled in liquid nitrogen and homogenized with a mill (Model A11B5001, IKA-Werk GmbH & Co. KG, Staufen, Germany) which had been cooled in liquid nitrogen and the powder was again homogenized mortar and pestle which had been cooled in liquid nitrogen. About fifty mg of the powder was homogenized with 100 μl of GS. The crude enzyme extract used for zymogram was prepared in the same procedures as those for the preparation of the enzyme extract from developing kernels, as described above.

### Native-PAGE/Activity Staining of BE Isozymes in Rice Endosperm and Culm

Activities of three BE isozymes (BEIIb, BEIIa, and BEI) in the crude enzyme extracts from developing endosperm and culms of Kinmaze and EM10 were determined according to the procedures described by Yamanouchi and Nakamura (1992).

### Preparation of Starch Granules in Rice Endosperm and Culm

About 25 mature rice kernels randomly selected from developing kernels harvested and pooled from about 5–10 individual plants were dehulled and their embryos were removed with forceps. The treated kernels were soaked in 30 ml of 0.1% (w/w) NaOH solution at 4°C for about 20 h. The kernels were washed seven times with 40 ml of distilled water and they were homogenized with mortar and pestle. The homogenate in 30 ml distilled water was filtered through nylon net with a pore size of 100 μm. The filtrate was centrifuged at 3,000 *g* for 20 min at 10°C. The precipitate was added by 30 ml of distilled water and mixed. The mixture was centrifuged at 3,000 *g* for 20 min at 10°C. The procedure was repeated with six times, and the washed precipitate was suspended with 30 ml of 10% (v/v) ethanol.

Three randomly chosen culms which had been harvested from three individual rice plants of Kinmaze and EM10 were cut into small pieces with scissors, cooled in liquid nitrogen, and homogenized with a mortar and pestle which had been cooled in liquid nitrogen. The resulting powered samples were suspended with 20 ml of 10% (v/v) ethanol. The homogenate was filtered through nylon net (pore size, 100 μm). The filtrate was centrifuged at 3,000 *g* for 20 min at 20°C. The precipitate was added by 1.6 ml of 90% (v/v) ethanol and mixed. The mixture was centrifuged at 3,000 *g* for 10 min at 20°C. The precipitate was suspended with 1 ml of distilled water and an aliquot (0.4 ml) was layered onto 1.3 ml of Percoll (MP Biomedicals, LLC., Illkirch, France) solution in a plastic tube. The tube was centrifuged at 3,000 *g* for 30 min at 20°C. The precipitate was suspended with 150 μl of 10% ethanol and stored at 4°C until used.

### Observation of Starch Granule Morphology in Rice Endosperm and Culm From a *be2b* Mutant Line EM10 and Its Wild-Type Cultivar Kinmaze

Scanning electron micrographs (SEM) of starch granules from endosperm and culm of rice Kinmaze and EM10 were captured with a field emission SEM (FE-SEM, SU-8010, Hitachi Hi-Technologies Corporation, Tokyo, Japan), as described previously (Nakamura et al., 2019). The starch granules from mature kernels and culms were placed on a double-sided *carbon* adhesive *tape* attached on the SEM sample holder and dried under air at room temperature overnight. The starch samples were coated with gold-palladium using a sputter coating device (Ves-10, Multi Coating Unit, Vacuum Device Inc., Mito, Japan) and their morphology was examined by FE-SEM.

### Analysis of Chain-Length Distribution of Amylopectin

The chain length distribution was determined by the fluorophore-assisted carbohydrate electrophoresis (FACE) method (O’Shea et al., 1998). The starch granules were suspended in distilled water (3 mg/ml) and heated at 100°C for 5 min and stood at room temperature. An aliquot of the glucan solution (0.06 mg) was taken and incubated with 12 mM sodium acetate buffer (pH 4.4) and *Pseudomonas amyloderamosa* isoamylase (PaISA, 100 units, Hayashibara Co., Ltd., Okayama, Japan) in a total volume of 200 μl at 37°C for 5 h, heated at 100°C for 5 min, and stood at room temperature. The cooled sample was deionized by mixing with 5 mg of AG 501-X8 (D) Resin (20–50 mesh, Bio-Rad) for 60 min at room temperature. The deionized sample was dried under vacuum. The reducing ends of the resulting linear glucans were labeled with a fluorescent probe, 8-amino-1,3,6-pyrenesulfonic acid (APTS, AB SCINEX, Tokyo, Japan) according to the instruction manual (AB SCINEX). The APTS-labeled linear glucan chains were separated and quantified by a laser-induced fluorescence detector (P/ACE MDQ Carbohydrate System, Beckman Coulter/AB SCINEX). The chain-length distribution of each glucan sample was measured three times.

### Analysis of Chain-Length Distribution of Phosphorylase (Φ)-Limit Amylopectin

The heated glucans (0.09 mg) were incubated in 80 mM Na-phosphate buffer (pH 6.8), rabbit muscle phosphorylase a (0.14 units; SIGMA), and 0.2 mM dithiothreitol in a total volume of 200 μl at 37°C for 6 h, followed by the addition of 0.14 units of phosphorylase a, then incubated at 37°C overnight. The samples were heated at 100°C for 5 min, and stood at room temperature. The resulting α-glucans were debranched by PaISA, deionized, and labeled with APTS, as described above. The chain length distribution of the resulting phosphorylase limit dextrins (Φ-LD) was determined by the FACE method as described above.

### X-Ray Diffraction Patterns

Wide angle X-ray diffraction (XRD) patterns of starch granules from mature endosperm of Kinmaze and EM10 were measured using a Rigaku RINT2500H/PC System X-ray diffractometer (Rigaku Corporation, Tokyo). The dry starch sample (ca. 100 mg) was soaked for at least 5 min and saturated with water. Samples were exposed to CuKα radiation (0.15418 nm) and scanned between 2θ = 2° and 40° at a scan rate of 2°/min with a step size of 0.02°. A current of 200 mA and voltage of 40 kV were used. The XRD apparatus had a divergence slit of 1°, a receiving slit of 0.3 mm, and a scatter slit of 1°.

### X-Ray Scattering Pattern by SAXS

Small-angle X-ray scattering (SAXS) measurements were performed with a Nano-viewer (Rigaku Co., Tokyo, Japan) using a Cu–Kα radiation source. The wavelength of the X-ray beam used was 0.154 nm, and the scattered X-rays were detected by a 2D detector (Pilatus 100K; Detris, Baden, Switzerland). The scattering vector, *q*, was recorded between 0.1 and 1.0 nm^–1^, where *q* is given by *q* = 4πsinθ/λ (2θ is the scattering angle). The sample was approximately 30 mg for all experiments. The specimen was placed between 50-μm-thick Kapton windows. The dried samples were the starch granules dried in vacuum oven at the room temperature. The wetted ones were the starch granules putting into the distilled water for 24 h.

### Solid State NMR Spectroscopy

All ^13^C-CPMAS spectra were measured at 125.77 MHz (Bruker Avance 500 spectrometer), with a CP-contact time of 10 ms with the size of FID of 2048 transients. A standard Bruker 4 mm BL4 MAS Probe spinning at 8 kHz was used, with a typical *π*/2-pulse length of 4 μs and a recycle delay of 5 s. The reference was tetramethyl silane (TMS) with glycine as a secondary standard (176.03 ppm for ^13^C). All spectra were taken at room temperature.

### Optical Sum Frequency Generation (SFG) Spectroscopy

For scattered SFG measurement the powder samples of EM10 and Kinmaze were put in transparent silica glass square cells (AS ONE Q-101) of sizes 3.5 mm × 12.5 mm × 45 mm. The internal sizes of the cells were 1mm in thickness and 10mm in width. The SFG spectroscopy system was already described previously (Hieu et al., 2015). Tunable infrared light pulses at wavelength of ∼3 μm was output from an optical parametric generator (EKSPLA PG401/DFG2-18P) pumped by the fundamental and third harmonic output of a Nd^3+^:YAG laser (EKAPLA PL2143B) with time width 30 ps and repetition rate of 10 Hz. The pulse energy of the visible light was from 5 to 10 μJ and that of the infrared (IR) was about 230 μJ at the sample. The spectral width of the IR light was 6 cm^–1^. The visible and infrared beams illuminated the sample with incident angles of 75° and 45°, respectively, with respect to the normal to the glass cell window plane. The SFG light was collected at the reflective angle of 70°. Near the entrance slit of the monochromator, band pass filters (Asahi SV0490) were put to block the excitation visible and infrared light beams. SFG spectra were taken from wavenumbers 2750 cm^–1^ to 3150 cm^–1^ with a scanning step of 10 cm^–1^. The accumulation at each wavenumber was done for 300 laser shots. The SFG signal of the GaAs reference sample was used to normalize the SFG of the sample. The polarization combination was p-polarization for SFG, p-polarization for visible and p-polarization for IR light (PPP) with respect to the incident plane.

## Results

### Native-PAGE/Activity Staining of BE Isozymes in Rice Endosperm and Culm

**FIGURE 1:**
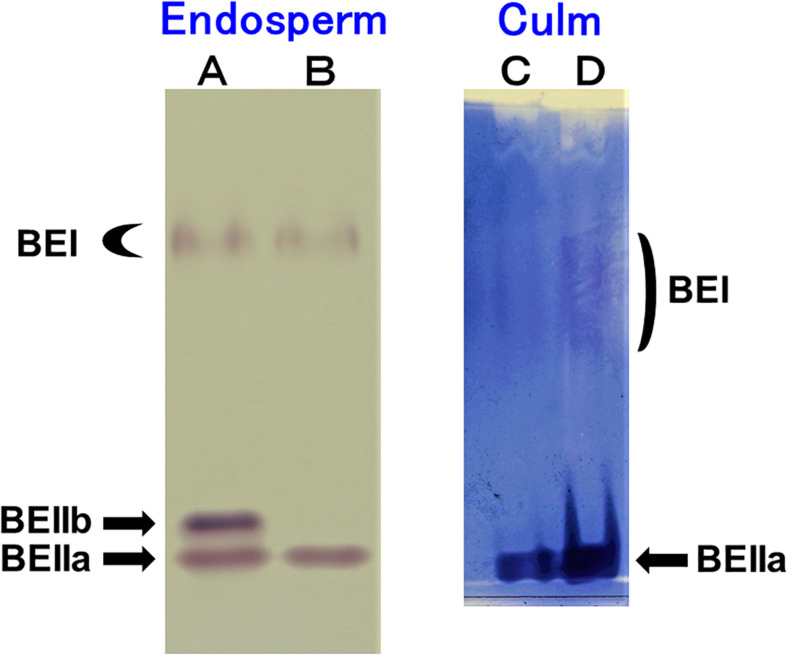
Native-PAGE/activity staining of BE isozymes in developing endosperms of Kinmaze and EM10 and culm from Kinmaze. **(A)** Kinmaze endosperm (10 μl of the crude enzyme extract); **(B)** EM10 endosperm (10 μl); **(C,D)** Kinmaze culm (6 and 12 μl). The experiments were repeated at least three times until all these results were consistent, whereas each figure shows one representative result.

The native-PAGE/activity staining method provides a useful information on the relative activities of BEI, BEIIa, and BEIIb in various rice organs (Yamanouchi and Nakamura, 1992). Figure 1A shows that three BE isozymes were present in developing endosperm of Kinmaze, whereas in EM10 the BEIIb activity was missing although BEI and BEIIa activities were unaffected. On the other hand, BEIIa accounted for the major BE activity in the culm of Kinmaze while the BEI activity was significantly present, but greatly lower than the BEIIa activity (Figure 1B). As it is known that BEIIb is specifically expressed in the endosperm of rice (Yamanouchi and Nakamura, 1992), the similar pattern of BE activities was found in the culm between EM10 and Kinmaze (data not shown). These differences in relative activities among three BE isozymes between culm and endosperm of WT and between WT endosperm and *ae* endosperm might reflect on differences in the amylopectin fine structure and starch granule crystalline structures.

### Changes in Morphology of Starch Granules in Endosperm and Culm of a *be2b* Mutant Compared With That of Its Parent Cultivar Kinmaze

**FIGURE 2:**
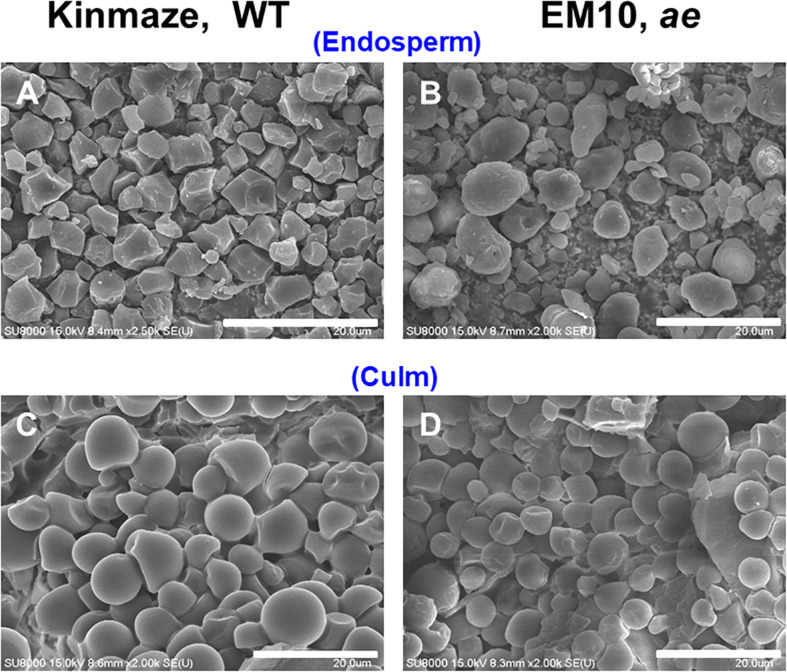
Scanning electron micrographs of starch granules in mature kernels and culms from a *be2b* mutant line, EM10 and its host wild-type *japonica* cultivar Kinmaze. **(A)** Endosperm starch granules from Kinmaze; **(B)** Endosperm starch granules from EM10; **(C)** Culm starch granules from Kinmaze; **(D)** Culm starch granules from EM10. Bars, 20 μm.

Starch granules in mature rice endosperm were polyhedrons with sharp edges ranging in size approximately from 3 to 10 μm (Figure 2A). The loss of BEIIb activity had a profound effect on the morphology of starch granules. Figure 2B shows that starch granules in mature *ae* mutant endosperm had irregular shapes and sizes with loss of sharp edges and most of them were smaller than WT granules. The result is consistent with observations of starch granules in *ae* mutants or transformants generated from *japonica*-type and *indica*-type rice (Nishi et al., 2001; Wei et al., 2010a; Butardo et al., 2011).

Culm or stem in rice plants is known to support the productivity of the seeds because the organ stores a good amount of starch granules before the anthesis, but after the anthesis these starches quickly degrade to synthesize sucrose and the sucrose is then translocated to the kernels (Horie et al., 2005). Starch granules from culm of the WT cultivar Kinmaze were frequently spherical whereas the size was similar to the polygonal endosperm granules (Figure 2C). Starch granules from culm of the *ae* mutant line EM10 resembled those of WT in both size and morphology (Figure 2D). It seemed to make sense if we consider that BEIIb was not expressed in the culm (Figure 1B) and hence the relative activities and composition of starch biosynthetic isozymes might be the same between Kinmaze and EM10.

### Comparison of Chain-Length Distribution of Amylopectin in Endosperm of a *be2b* Mutant With That of Its Parent Cultivar Kinmaze

To examine the contribution of BEIIb to the fine structure of amylopectin, we determined the chain-length distribution of amylopectin in mature kernels in a *be2b* mutant line EM10 and its parent cultivar Kinmaze after debranching the insoluble glucans with PaISA, followed by labeling of liberated chains with APTS at their non-reducing ends, according to the FACE method (O’Shea et al., 1998). Thus, in this study the distribution of α-1,4 chains of amylopectin was analyzed on molar basis.

**FIGURE 3:**
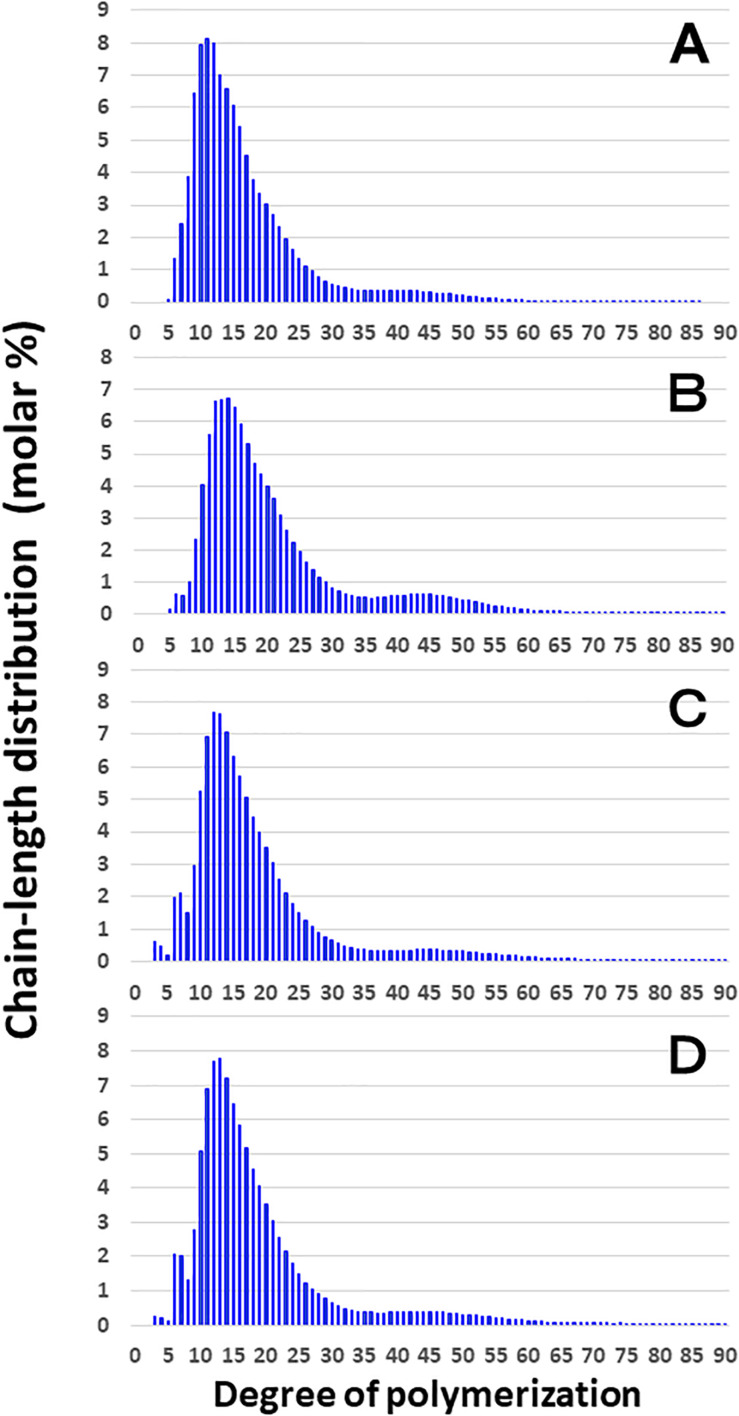
Chain-length distribution of amylopectin and its phosphorylase-limit dextrin in mature endosperm and culm from a *be2b* mutant line, EM10 and the host wild-type *japonica* cultivar Kinmaze. The vertical axis presents the proportion (molar %) of the amount of each chain to the total amounts of chains with degree of polymerization (DP) from 3 to 90 whereas the horizontal axis shows the DP value of the chain. **(A)** Endosperm starch granules from Kinmaze; **(B)** Endosperm starch granules from EM10; **(C)** Culm starch granules from Kinmaze; **(D)** Culm starch granules from EM10. The experiments were repeated at least three times until all these results were consistent, whereas each figure shows one representative result. Values are the averages calculated from three replicate measurements. Standard deviations were too small to be shown in the figure.

**FIGURE 4:**
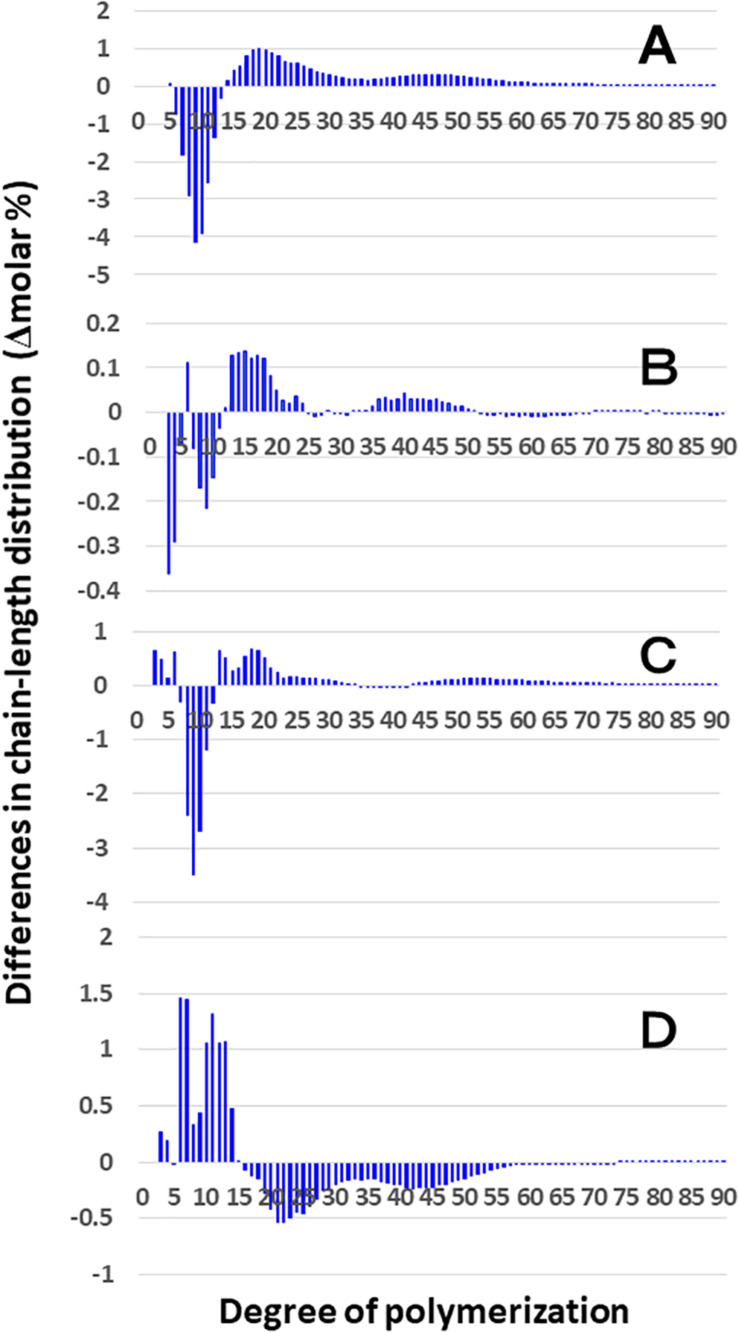
Difference in chain-length distributions of amylopectin from each *be2b* mutant line, EM10 compared to Kinmaze. Data are the same as those shown in Figure 3. The other conditions are the same as in Figure 3. **(A)** Data for amylopectin of the EM10 endosperm subtracted by those of the Kinmaze endosperm. **(B)** Data for amylopectin of the EM10 culm subtracted by those of the Kinmaze culm. **(C)** Data for amylopectin of the Kinmaze culm subtracted by those of the Kinmaze endosperm. **(D)** Data for amylopectin of the EM10 culm subtracted by those of the EM10 endosperm.

Figure 3 shows that the chain length distributions of insoluble glucans in the kernels and culms of both Kinmaze and EM10 exhibited the bimodal patterns, indicating that these glucans have the amylopectin-type cluster structure. However, the fine structure of amylopectin in the endosperm of EM10 greatly differed from that of Kinmaze. The proportion of long chains which comprised of the cluster interconnecting B2 and B3 chains with degree of polymerization (DP) of longer than about 37 was markedly higher in EM10 than that in Kinmaze (Compare Figure 3B with Figure 3A). The plot showing the difference in amylopectin chain length distribution between EM10 and Kinmaze indicates that short chains of DP about 6–13 with a peak around DP9-10 dramatically decreased whereas intermediate and long chains of DP ≥ 15 clearly increased in EM10 compared with Kinmaze (Figure 4A). The chain-length distribution of *be2b*-amylopectin in rice endosperm was also analyzed using other allelic *be2b*-mutant lines, EM72 and EM224. The Supplementary Figure S1 shows that the chain-length profiles in amylopectin of both mutants were quite similar to that of EM10, indicating that the phenotype in EM10 was common in all *be2b*-mutants.

These results indicate that the *be2b* mutation increased the average length of chains of amylopectin, while it decreased the number of chains per cluster because the ratio of amounts of A-chains plus B1 chains to those of B2-3 chains decreased in the *ae* mutant.

### Comparison of Chain-Length Distribution of Amylopectin in Culm of a *be2b* Mutant With That of Its Parent Cultivar Kinmaze

On the other hand, no significant difference in amylopectin in the culm was found between EM10 and Kinmaze (Figures 3C,D). When the chain profile of the Kinmaze amylopectin in the endosperm was compared with that in the culm (Figure 4C), the similar trend induced by loss of BEIIb in the endosperm was observed. The finding that in the culm short chains of DP7–12 significantly depleted while intermediate and long chains of DP ≥ 13 were enriched than those in the endosperm (Figure 4C) suggests that BEIIb was missing in the culm. The idea is consistent with the results shown in Figure 1B and with the reports indicating that BEIIb activity is greatly depressed in the culm (Yamanouchi and Nakamura, 1992) and that the *BEIIb* transcript is specifically expressed in the endosperm in rice (Ohdan et al., 2005).

Figure 4D shows that in EM10 the culm amylopectin had more short chains of DP ≤ 14 and less intermediate and long chains of DP ≥ 16 compared with the endosperm amylopectin in spite of absence of BEIIb in both tissues. The result suggests that other factor(s) supplemented the function of BEIIb in the culm.

### Comparison of Chain-Length Distribution of Phosphorylase-Limit Dextrins (ΦLD) of Amylopectin in Culm of a *be2b* Mutant With That of Its Parent Cultivar Kinmaze

For the purpose of analysis of the inner structure of amylopectin, it was treated with phosphorylase a from rabbit muscle to cleave outer non-branched segments up to DP4 from the non-reducing ends of amylopectin chains. After the treatment, the resulting DP4 chains are almost derived from A chains whereas the other chains of DP ≥ 5 are derived from B and C chains (Bertoft, 2015).

**FIGURE 5:**
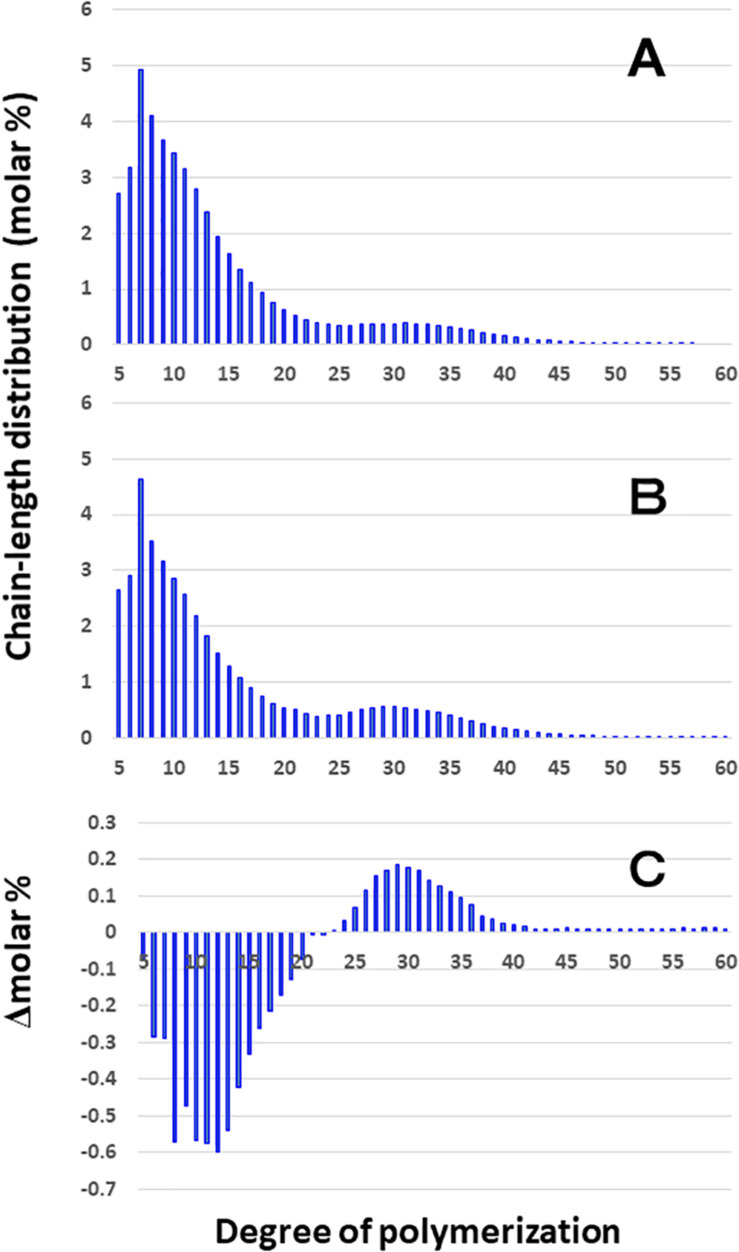
Difference in chain-length distributions of phosphorylase-limit dextrins (Φ-LD) of amylopectin from each *be2b* mutant line, EM10 compared to Kinmaze. The conditions are the same as those shown in Figure 3, whereas data are shown from DP5 to DP60. **(A)** Data for Φ-LD of amylopectin of the Kinmaze endosperm. **(B)** Data for Φ-LD of amylopectin of the EM10 endosperm. **(C)** Data for Φ-LD of amylopectin of the EM10 endosperm subtracted by those of the Kinmaze endosperm.

Although the difference in chain-length of Φ-LD between EM10 and Kinmaze in the culm was much smaller than that in the endosperm, the *be2b* mutation slightly induced the decrease in the DP5–20 chains and increase in the DP ≥ 24 chains in the culm (Figure 5). The results are consistent with the view that BEIIb was responsible for the synthesis of B1 chains as well as A chains, but not the synthesis B2-3 chains.

### X-Ray Diffraction Patterns

**FIGURE 6:**
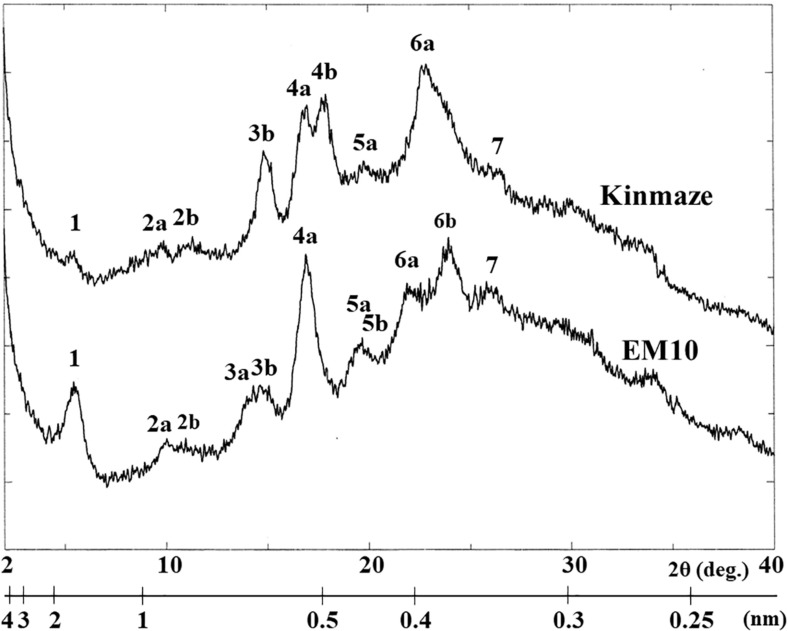
Wide angle X-ray diffraction analysis of starch granules in mature kernels from a *be2b* mutant line, EM10 and its host wild-type *japonica* cultivar Kinmaze. The experiments were repeated at least three times until all these results were consistent, whereas each figure shows one representative result.

X-ray diffraction is the analytical method determining the atomic and molecular structure of a starch crystal, in which the crystalline structure causes a beam of incident X-rays to diffract into many specific directions. Katz and van Itallie (1930) reported that starch granules show three crystalline polymorphs, namely, A-type, B-type, and C-type. Starch granules in mature endosperm of Kinmaze displayed X-ray diffraction peaks at 2*θ* of approximately 14.91° (a single peak, 3b), 16.97° and 17.92° (doublet peaks, 4a and 4b), and 22.89° (a single peak, 6a) (Figure 6 and Table 1), which are characteristics of A-type starch granules in cereal endosperm. A small peak at 2*θ* of approximately 5.49° (a single peak 1), which is one of characteristics for B-type starch, suggests that Kinmaze starch was classified into the C-type starch which mainly contained the A-type starch component with a small part of the B-type starch component.

**TABLE 1:** Parameters of wide angle X-ray diffraction analysis of starch granules in mature kernels from a *be2b* mutant line, EM10 and its host wild-type *japonica* cultivar Kinmaze.

Peak number	*d* (Å)	2*θ* (degree)	*d* (Å)	2*θ* (degree)
			
	Kinmaze (WT)		EM10 (*ae*)	
1	16.10	5.49	16.04	5.51
2a	8.98	9.85	8.84	10.01
2b	7.83	11.30	8.08	10.95
3a			6.20	14.28
3b	5.94	14.91	6.04	14.66
4a	5.22	16.97	5.24	16.93
4b	4.95	17.92		
5a	4.47	19.88	4.55	19.52
5b			4.44	19.98
6a	3.89	22.89	4.05	21.97
6b			3.71	23.96
7	3.37	26.47	3.45	25.86

**d*-value, the spacing between diffraction planes, was calculated according to Bragg’s law (*d* = 2π/*θ*) by assuming that the wavelength of the CuKα beam is 1.5418 Å. *θ*, half the scattering angle.*

Starch granules in mature EM10 endosperm showed the characteristic diffraction peaks at 2*θ* of approximately 5.51° (a single peak, 1), 16.93° (a single peak, 4a), 21.97° and 23.96° (doublet peaks, 6a and 6b) (Figure 6 and Table 1). Since these peaks are commonly detected those in the B-type starch, the present result indicated that loss of BEIIb activity resulted in the synthesis of B-type starch in the endosperm.

### X-Ray Scattering Pattern by SAXS

**FIGURE 7:**
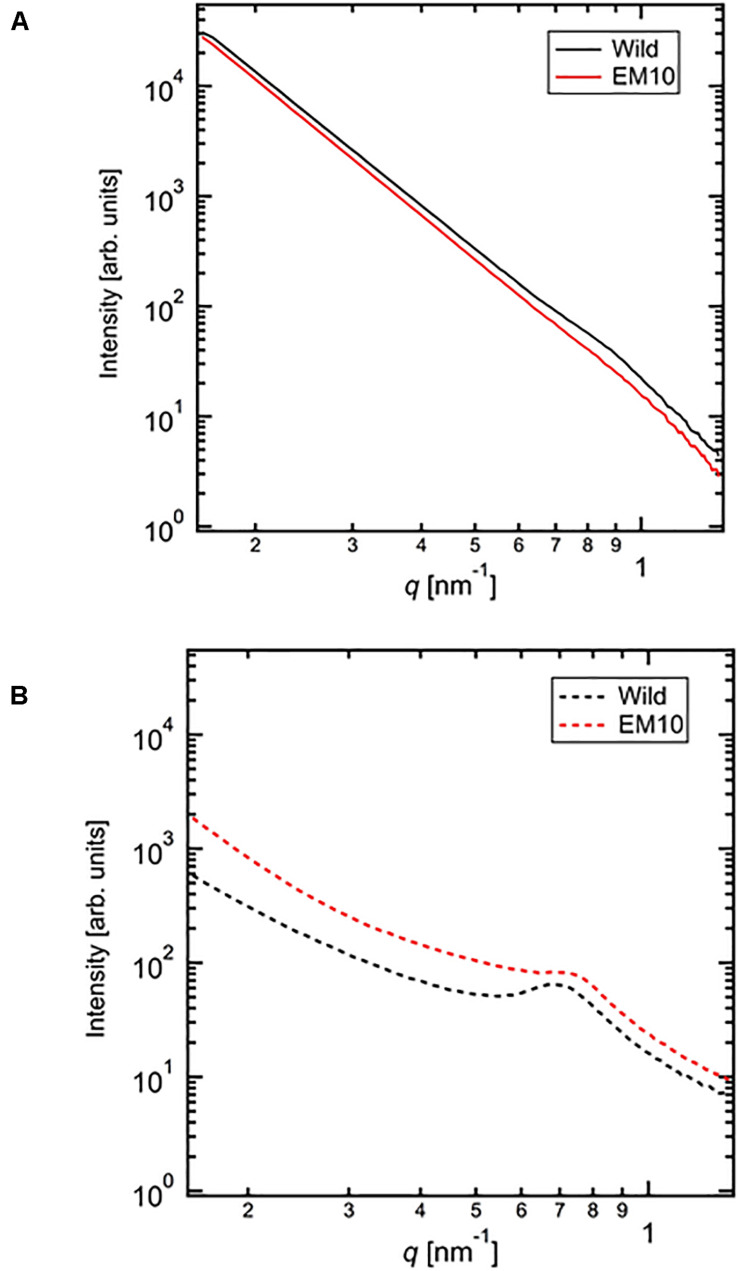
Small angle X-ray scattering (SAXS) analysis of starch granules in mature kernels from a *be2b* mutant line, EM10 and its host wild-type *japonica* cultivar Kinmaze. The experiments were repeated at least three times until all these results were consistent, whereas each figure shows one representative result using the dried sample **(A)** or the wetted one **(B)**. The other conditions are the same as in Figure 6.

The SAXS profiles of the dried starches from mature endosperm of WT Kinmaze and EM10 (Figure 7A) and the wetted starches in Kinmaze and EM10 (Figure 7B). For all samples, the SAXS pattern indicated a peak at *q*_*peak*_ due to the correlation length between crystals (*d* = 2π/*q*_*peak*_). The correlation length was estimated to be 7.32 nm in dried Kinmaze starches (Figure 7A). On the other hand, no peaks were observed in the case of dried EM10. This result suggests that the *ae*-amylopectin branches were composed to the nematic phase (Donald et al., 2001). The starches from wide-type Kinmaze had density fluctuations due to their presence in the smectic phase, keeping the so-called “9 nm-repeat structure,” while the dried EM10 starches were mainly in the nematic phase, and then no density fluctuations were observed.

We evaluated the correlation length of the wetted samples (Figure 7B). Both samples were observed the correlation peaks due to 9 nm-repeat. The length was 8.81 nm for wetted wild type starch while it was 8.24 nm for wetted EM10 starch. The increase of the correlation after wetting with the water molecules in WT Kinmaze would be caused by absorption of water into the amorphous region between crystals of starch granules. Blazek and Gilbert (2011) reported that, in the presence of water, plasticization of amylopectin branch points allows the double helices to be decoupled from the polymer backbone and aligned into lamellar register, resulting in a transition from the nematic to the smectic phases. Therefore, in the EM10 starches, after wetting, the correlation between amorphous and crystal regions was observed clearly because of transition from nematic to smectic phases. Furthermore, both intensity peaks were stronger due to enlargement of density fluctuations. These suggests that water molecules were absorbed mainly into the amorphous region. However, the peak intensity of EM10 was much weaker than that of WT Kinmaze. From the result of X-ray diffraction, the crystal form of EM10 starch was the B-type which included more water molecules in the crystal lattice than the A-type (Imberty et al., 1991), then the density fluctuations became weaker due to inclusion of more water molecules in both amorphous and crystal region.

### Solid State NMR Spectroscopy

**FIGURE 8:**
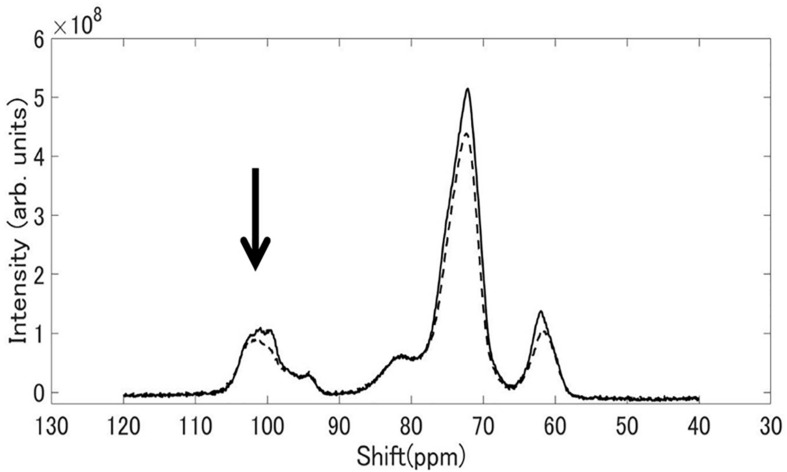
^13^C CP/MAS NMR spectra of starch granules in mature kernels from a *be2b* mutant line, EM10 (dashed curve) and its host wild-type *japonica* cultivar Kinmaze (solid curve). The experiments were repeated at least three times until all these results were consistent, whereas each figure shows one representative result. The other conditions are the same as in Figure 6.

Figure 8 shows ^13^C CP / MAS NMR spectra of starch granules purified from mature rice kernels of EM10 and Kinmaze. Peaks were seen at the chemical shift of 62, 72, and 100 ppm. The shapes of the structures around 100 ppm differed remarkably between EM10 and Kinmaze. It is known that the peak around 100 ppm is assigned to the C-1 carbon of glucose (Gidley and Bociek, 1985). If this peak is composed of two small peaks (100.0, 99.2 ppm), it is assigned to the B-type starch, while if it is composed of three peaks (100.4, 99.2, 98.2 ppm), it is assigned to the type A-type starch (Gidley and Bociek, 1985; Tan et al., 2007). Based on this criterion, starches in mature endosperm from Kinmaze and EM10 were considered to be the A-type and the B-type, respectively, because the former exhibited the broad peaks at around 100 ppm whereas the latter showed one asymmetric peak (Figure 8).

### Optical Sum Frequency Generation (SFG) Spectroscopy

**FIGURE 9:**
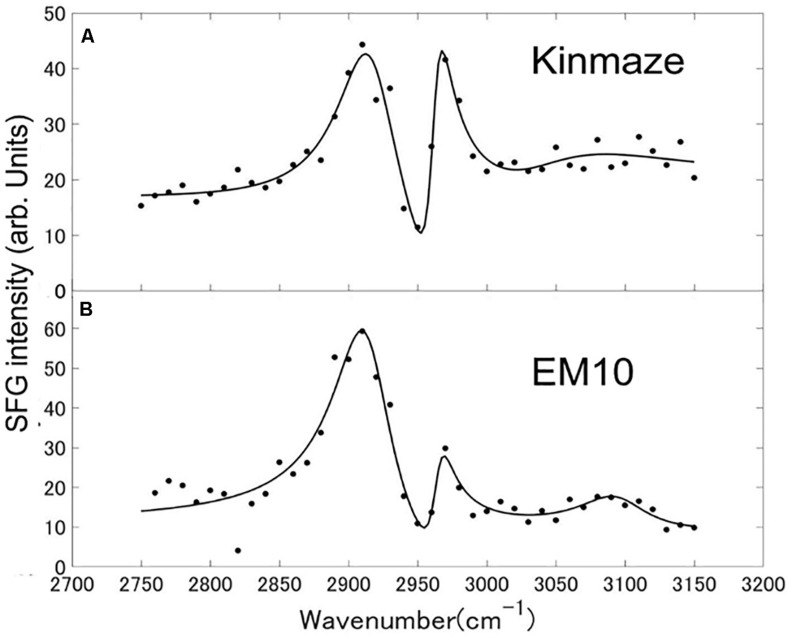
SFG spectra of starch granules in mature kernels from a *be2b* mutant line, EM10 **(B)** and its host wild-type *japonica* cultivar Kinmaze **(A)**. The experiments were repeated at least three times until all these results were consistent, whereas each figure shows one representative result. The other conditions are the same as in Figure 6.

Sum frequency generation spectra of starch powder samples purified from mature Kinmaze and EM10 kernels were measured in the wavenumber ranging from 2750 to 3150 cm^–1^, as shown in Figure 9. The incident visible light, infrared light, and SFG light were all p-polarizations. The efficiency change of the measurement system as a function of the wavelength was compensated for by using a GaAs (001) crystalline wafer as a reference sample and normalizing the SFG intensity spectrum of the target samples. Peaks were seen at wavenumbers of around 2910 cm^–1^, 2970 cm^–1^, and 3100 cm^–1^, although the relative intensities of these peaks were different between EM10 and Kinmaze. The Kinmaze starch showed the two peaks almost at the same height (Figure 9A), while in the EM10 starch the height of the C-H_2_ peak at 2970 cm^−1^ was about 40% of that of the C-H peak at 2910 cm^−1^ (Figure 9B). Previously, Kong et al. (2014) reported that the heights of the two peaks in the SFG spectra of A-type starch are almost the same, while B-type starch gives a smaller peak intensity on the high frequency side. Based on this criterion, the present results confirmed that the starches of EM10 and Kinmaze were of B- and A-types, respectively, consistent with the conclusion obtained from the results by NMR (Figure 8).

## Discussion

Effects of loss of BEIIb activity on the amylopectin fine structure and starch granular structures and physicochemical/functional properties have been examined in a variety of cereals. The BEIIb deficient mutants (often called *ae* mutants) and transformed lines have been extensively examined because they have mostly dull and often shriveled kernels which accumulate greatly modified starches such as higher amylose content, higher onset temperature for gelatinization, and higher resistant starch content (see reviews by Shannon et al., 2009; Regina et al., 2015; Tetlow and Emes, 2017; Nakamura, 2018). It is widely accepted that the distinct structure of *ae* amylopectin is responsible for the modification of starch granular structures in endosperms of maize (Brown et al., 1971; Li et al., 2007) and rice (Nishi et al., 2001; Tanaka et al., 2004; Nakata et al., 2017), and these phenomena were proved by using transformed lines in which the expression of BEII-type isozyme(s) was(were) silenced in endosperms of rice (Wei et al., 2010a,b; Butardo et al., 2011; Man et al., 2013; Sawada et al., 2018), wheat (Regina et al., 2005), and barley (Regina et al., 2010). *In vitro* studies with purified BEIIb have characterized its enzymatic properties, e.g., a highly preference for short chains compared with BEI from maize (Guan and Preiss, 1993; Takeda et al., 1993) and rice (Nakamura et al., 2010; Sawada et al., 2014).

### Contributions of BEIIb to the Fine Structure of Amylopectin in Rice Endosperm

In the present study, the structural features of amylopectin in the *ae* mutant of rice were analyzed. The higher proportion of the B2-B3 chains of DP ≥ 37 in amylopectin from the *ae* endosperm compared with that from the WT endosperm (Figures 3A,B, 4A) indicates that the number of chains per single cluster (# of all chains/# of B2-B3 chains) was smaller in *ae* amylopectin. In other word, this means that BEIIb was involved in the synthesis of A chains and B1 chains, but not in that of B2-B3 chains. The finding that short chains of DP6-14 decreased while intermediate chains of DP15-32 increased in *ae* amylopectin (Figure 4A) indicates that BEIIb preferentially and specifically synthesized A chains rather than B1 chains, and therefore that in the absence of BEIIb activity the average length of chains in amylopectin was longer than that in WT amylopectin. In summary, BEIIb is considered to play a distinct role in the formation of new A chains of amylopectin cluster by attacking exterior B1 chains and A chains of the amylopectin cluster in rice endosperm.

Chain-length distribution analysis of Φ-LD can provide us with information on the inner structure of amylopectin fine structure (Bertoft, 2015). The result that the Φ-LD of *ae*-amylopectin had fewer short and intermediate chains of DP5-20 and more long chains of DP24-40 than that of WT amylopectin (Figure 5) confirmed that BEIIb preferentially attacked A and B1 chains, but was unable to react to B2-B3 chains.

**FIGURE 10:**
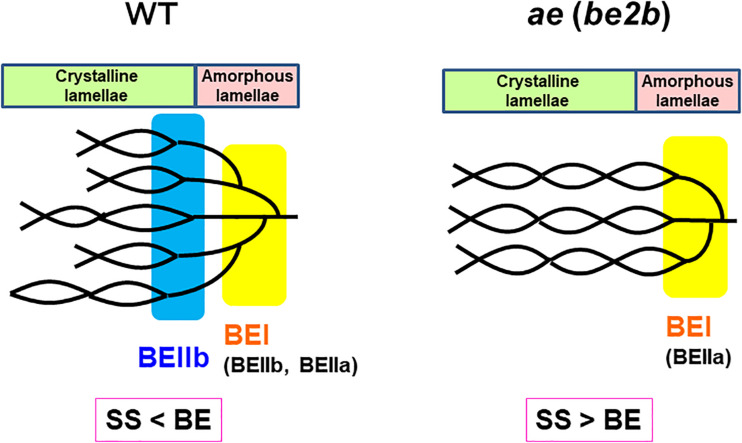
A schematic representation of the hypothetical cluster structure of amylopectin in endosperm of the wild-type *japonica* rice compared with that of a *be2b* (*ae*) mutant. According to the past and present studies, we hypothesize that branch points are located in two separate zones which are mainly formed by BEI and BEIIb, respectively. The first branch points are localized in the amorphous lamellae whereas the second branch points are localized in the reducing part of the crystalline lamellae, and presumably, in addition the intermediate region between both lamellae. Note that the relative SS activities versus BE activities differ between in the wild-type and *be2b* mutant endosperms and this affects the average length of chains. In the wild-type endosperm, due to the SSIIa deficiency in *japonica* rice (Nakamura et al., 2005), the relative SS activity for elongation of cluster chains vs. the BE activity is relatively low, and this results in the shorter chain-length of the cluster chains. On the other hand, in the *be2b* mutant the ratio of SS activity to BE activity becomes higher, and this causes the average chain-length of cluster chains to be longer than that of wild-type. This model has some similarity to that proposed by Jane et al. (1997) and illustrated by Yoo and Jane (2002) that the branch points in A-type amylopectin are scattered and located in both amorphous and crystalline lamellae while those in B-type amylopectin are mostly clustered in the amorphous lamellae. However, the present model clearly differs from their model in that branch points in wild-type amylopectin (A-type) in Kinmaze are localized in the restricted zone of the crystal lamellae and intermediate between the crystalline and amorphous lamellae, but not widely distributed (compare Figure 10 in this paper with Figure 4 by Yoo and Jane, 2002).

Based on these results, the structural differences of amylopectin between the WT *japonica*-type rice and the *ae* mutant can be described. First, the average length of A and B1 chains was longer in *ae* amylopectin than WT amylopectin (Figure 4A). Second, the number of A chains plus B1 chains per single cluster was smaller in *ae* amylopectin than WT amylopectin (Figure 4A). Third, the average length of double helices must be much longer in *ae* amylopectin compared with WT amylopectin because it is known that the onset temperature of gelatinization in *ae* amylopectin from EM10 is markedly higher than that in WT amylopectin from Kinmaze (Nishi et al., 2001). A dramatic change in the fine structure of amylopectin in the *ae* mutant endosperm was supported by the present results that the crystalline structures of starch granules in endosperm of EM10 were clearly different from those of Kinmaze (Figures 6–9 and Table 1). Based on the present and past investigations, the difference of amylopectin structure between Kinmaze and EM10 is schematically illustrated in Figure 10. In this model, during the synthesis of the new cluster, the initial branches are formed mainly by BEI in the basal region of the cluster, although BEIIb and BEIIa can contribute to this reaction to some extent. The idea was supported from the experimental results with the *be1* mutant, showing that the specificity of BEI for the formation of intermediate chains is not high, and this role can be significantly supplemented by BEIIb and/or BEIIa (Nakamura, 2002; Satoh et al., 2003). The analysis of *in vitro* BE reaction products also showed that BEI synthesizes preferentially intermediate chains rather than short chains whereas BEIIb and BEIIa prefer to form short chains, but scarcely form intermediate chains (Nakamura et al., 2010; Sawada et al., 2014). Figure 10 also hypothesizes that the role of BEIIb is to synthesize the new short chains forming the second branches, presumably in the restricted crystalline lamellae neighboring to the amorphous lamellae and the intermediate region between the amorphous lamellae and the crystalline lamellae. If the hypothesis is the case, it is interesting to ask the reason why the BEIIb can form the short chains in the restricted area. It is known that rice BEIIb has a very strict chain-length specificity because it synthesizes almost exclusively the DP7 and DP6 chains by attacking the DP12-14 chains (Nakamura et al., 2010; Sawada et al., 2014). It can be also pointed out the rice endosperm BEIIb plays a key role in the formation of enzyme-enzyme complexes (Crofts et al., 2015, 2017, 2018). These properties of BEIIb might be related to its role during the formation of cluster chains and hence BEIIb could affect profoundly the crystalline characteristics and physiochemical properties of starch granules.

### Difference in Structural Features of Amylopectin Between Endosperm and Culm of Rice and the Role of BEIIa in Amylopectin Biosynthesis in Both Organs

Since BEIIb was not or scarcely expressed, but instead BEIIa was highly expressed in the culm (Figure 1B), the contribution of BEIIa to the amylopectin structure could be detected by comparing the chain-length distribution of amylopectin from culm between WT and the *ae* mutant.

Considering that no BEIIb activity was detected in the culm even in the WT (Figures 1C,D), it is rational to find that there was no significant difference in amylopectin in the culm between EM10 and Kinmaze (Figures 3C,D, 4B). However, it is noted that the difference in amylopectin between the culm and the endosperm of WT (Figure 4C) was distinct from that in endosperm amylopectin between the *ae* mutant and WT (Figure 4A). Firstly, the extent of difference was smaller, and secondly, the pattern of change was different. The lengths of decreased short chains in the WT culm amylopectin compared with those in the WT endosperm amylopectin were DP8-11 (Figure 4C), whereas those in the *ae* endosperm amylopectin compared with the WT endosperm amylopectin were DP7-12 (Figure 4A). These results suggest that BEIIa played some role in the synthesis of A chains as well as some short B1 chains in the culm when BEIIb was lacking. Is there any scenario explaining the different contribution of BEIIa to amylopectin biosynthesis either in the presence or absence of BEIIb? As already mentioned above, Crofts et al. (2018) recently reported that the BEIIb protein must play an important role in the formation of several kinds of protein complexes among starch biosynthetic isozymes in rice endosperms using a *japonica*-type WT rice cultivar Nipponbare and EM10. For example, BEIIb forms a protein complex among BEI, SSI, SSIIa, and SSIIIa. Surprisingly, in EM10 the BEIIa protein can bind to BEI, SSI, and SSIIIa. In addition, the loss of BEIIb causes many isozymes including BEIIa, BEI, SSI, SSIIa, SSIIIa, phosphorylase1, and pullulanase to bind to starch granules. These phenomena suggest that BEIIb shifted the physiological state of BEIIa to “the active state” in its absence from “the inactive state” in its presence, although this hypothesis should be proved by future study.

### Relationship Between the Fine Structure of Amylopectin and the Internal Structure of Starch Granules

The present investigations established that the change in the fine structure of amylopectin caused by loss of BEIIb activity profoundly affected the crystalline structures of starch granules in rice endosperm, as determined by using various methodologies.

First, the present analysis of the WRD peaks indicated that the WT starch granules from Kinmaze displayed an A-type scattering pattern, with an exception of a small peak at 2θ of approximately 16.10° (Figure 6 and Table 1), which is characteristic of a B-type starch. Thus, it is thought that the major starch granules in Kinmaze endosperm were the A-type, as usually found in cereal endosperm, whereas a small amount of B-type starch was included in Kinmaze endosperm. In contrast, in the *ae* mutant rice line EM10 the B-type starch granules were accumulated (Figure 6 and Table 1). The result is consistent with the previous reports with *ae* mutants of maize (Brown et al., 1971; He et al., 2020) and rice (Tanaka et al., 2004; Kubo et al., 2008; Wei et al., 2010b) and transformants where BEIIb activity was severely inhibited in rice (Butardo et al., 2011).

Second, SAXS analysis also revealed that features of the crystalline structures of starch granules in mature endosperm greatly differed between Kinmaze and EM10 (Figure 7). In the SAXS curve, the water molecules in the amorphous region would make correlation length expanding and density fluctuations emphasizing. The peak intensity of wild type was much stronger than that of EM10 in both dry and wet conditions. This suggests that the crystallinity of starches in EM10 was reduced by disturbance of the formation and/or arrangement of double helices due to scattered branch points mainly formed by BEI having a lower specificity for chain-length compared with BEIIb (O’Sullivan and Perez, 1999; Nakamura et al., 2010; Sawada et al., 2014). It is thought that the wetted WT starch had high crystallinity and more water absorbable amorphous region. The wetted EM10 starches would have smaller amorphous region, which resulted in reduction of the density fluctuations both in size and amplitude.

Third, although starch granules in Kinmaze and EM10 showed the similar ^13^C CP/MAS NMR spectra, there was a difference around the chemical shift of 100 ppm (Figure 8). These shapes of the peak(s) at around 100 ppm led us to the conclusion that Kinmaze and EM10 had the A-type starch and the B-type starch, according to the criteria established previously (Gidley and Bociek, 1985; Tan et al., 2007). The similar results were reported by several groups showing that the cereal starches were changed from A-type to B-type when BEIIb activity was lost in endosperms of rice (Wei et al., 2010b; Butardo et al., 2011) and maize (He et al., 2020).

Tan et al. (2007) reported that type V amorphous amylose shows peaks at 102 ppm and 82 ppm. Although the peak at 102 ppm overlaps with those of types A and B starch, each component can be separated by subtraction. The NMR spectrum of V amylose read from the chart reported by Tan et al. (2007) was subtracted from the spectrum of EM10 shown in Figure 8. The magnification of the V amylose data was adjusted so that the subtracted data became zero at around 83 ppm. The spectrum of EM10 became a one having two components superposed around 100.0 ppm, as shown in Supplementary Figure S2. This result was similar to that of the type B high amylose starch, as reported by Tan et al. (2007). Therefore, their result also supports our view that EM10 had the B-type starch granules in the endosperm.

Fourth, we compared the SFG spectra obtained using starch granules in mature endosperms from Kinmaze and EM10. Based on the relative intensities of the peaks at around 2910 cm^–1^ (the C-H peak) and 2970 cm^–1^ (the C-H_2_ peak), we could conclude that Kinmaze and EM10 had the A-type and B-type starches, respectively (Figure 9). These results are consistent with the previous report with acid-hydrolyzed maize high amylose (*ae*-type) starches Hylon VII (Kong et al., 2014).

Kong et al. (2014) suggested that the peak near 3100 cm^–1^ could be attributed to strongly hydrogen-bonded OH groups. Indeed, it is widely known that adsorbed and oriented H_2_O molecules show a peak near 3100 cm^–1^ in some condition (Shen and Ostroverkhov, 2006). Clear assignment of this peak is impossible at present due to the lack of sufficient information.

The two peaks in Figures 9A,B as a function of the wavenumbers from 2750 to 3150 cm^–1^ have been fitted to theoretical curves with three vibrational modes of Eq. (1) as below by the least square fitting method using a computer software Matlab,

$$\begin{array}{c} |\chi^{S\,F\,G}{|}^{2}=|\chi^{N\,R}+\frac{A_{1}\,\exp\left(i\,\theta_{1}\right)}{\omega -\omega_{1}+i\,\gamma_{1}}+\frac{A_{2}\,\exp\left(i\,\theta_{2}\right)}{\omega -\omega_{2}+i\,\gamma_{2}}+\frac{A_{3}\,\exp\left(i\,\theta_{3}\right)}{\omega -\omega_{3}+i\,\gamma_{3}}{|}^{2} \end{array}$$

In Eq. (1) χ^*SFG*^is a non-linear susceptibility of the SFG response and χ^*NR*^ is a background non-linear susceptibility. A_1_, A_2_, and A_3_ are constants, and θ_1_, θ_2_, and θ_3_ are phase differences of the three vibrational modes with respect toχ^*NR*^. ω_1_, ω_2_, and ω_3_ are resonance frequencies of the vibrational modes, and γ_1_, γ_2_, and γ_3_ are their resonance widths. Table 2 shows the fitting parameters used for the two spectra in Figures 9A,B.

**TABLE 2:** Parameters used for fitting the SFG spectra of starch granules in mature kernels from a *be2b* mutant line, EM10 and its host wild-type *japonica* cultivar Kinmaze to the theoretical curve in Eq. (1).

		Kinmaze (WT)	EM10 (*ae*)
Non-resonant susceptibility (arbitrary units)	χ^*NR*^	4.281	3.193
Amplitude (arbitrary units)	A1	88.18	128.4
	A2	32.58	22.24
	A3	57.86	33.13
Resonant wavenumber (cm^–1^)	ω_1_	2917	2915
	ω_2_	2962	2964
	ω_3_	3038	3095
Width (cm^–1^)	γ_1_	27.05	25.69
	γ_2_	8.619	8.500
	γ_3_	64.70	29.88
Phase (rad)	θ_1_	1.749	2.143
	θ_2_	0.1654	0.640
	θ_3_	0.001538	2.145

In Table 2 the difference ω_2_−ω_1_ between the two resonance frequencies of the C-H(ω_*1*_) and C-H_2_(ω_*2*_) modes was larger for EM10 than for Kinmaze only by 4 cm^–1^. The resonance widths γ_*1*_ and γ_*2*_ and phases θ_*1*_ and θ_*2*_ were not so different between EM10 and Kinmaze starches. On the other hand, the amplitude ratios A_1_/A_2_ were different; i.e., it was 5.8 and 2.7 for EM10 and Kinmaze starches, respectively (Table 2). Therefore, the large difference in the spectral shapes between them (Figures 9A,B) was dominated by the difference in the amplitudes A_1_ and A_2_ of the two vibrational modes. This point was not discussed by Kong et al. (2014), although the present SFG data are mostly consistent with theirs, assuming that Kinmaze and EM10 are A and B-type amylopectin, respectively. The reason for this variation of A_1_/A_2_ should be clarified by molecular dynamic calculation.

In summary, all the present analyses with XRD, SAXS, solid state NMR spectroscopy, and SFG are consistent that the endosperm starch from a *japonica*-type rice Kinmaze was shifted from the WT A-type to the B-type by the *ae* mutation. This change must have been caused by alteration of the fine structure of amylopectin in the *ae* mutant. The present experimental results suggest the way how the fine structure was altered by the *ae* mutation and this alteration could affect the parameters of these physical methodologies. Of particular interest is that the peak lengths of amylopectin clusters in wetted starch granules in EM10 endosperm (approximately 8.24 nm) was apparently shorter than those in Kinmaze endosperm (approximately 8.81 nm) (Figure 7B). On the other hand, the peaks of long chains (B2-3 chains) in chain-length distribution of endosperm amylopectin were approximately DP45 (Figures 3A,B), suggesting that the chain-length of a single cluster was unaffected by the absence of BEIIb activity. It is known that near the branch points of amylopectin cluster there are some regions where side chains cannot form double helices in parallel manners (O’Sullivan and Perez, 1999; Pérez and Bertoft, 2010). One possible explanation is that in the absence of BEIIb the branch points of *ae* amylopectin cluster were localized around the basal zone of the cluster forming the amorphous lamellae, whereas in WT amylopectin cluster branch points were distributed in wider zones than *ae* amylopectin cluster. This caused the apparent size of the cluster to be reduced in *ae* amylopectin in starch granules. This interpretation seems to consistent with the model illustrated in Figure 10, and basically match an idea proposal by Jane et al. (1997) that A-type amylopectin has branch points scattered in both amorphous and crystalline lamella whereas B-type amylopectin had most branch points localized in the amorphous lamellae. The idea seems to sharply contrast with results with maize *ae* and related mutants by Yao et al. (2004). They concluded that all the three BE isozymes share similar patterns in the initiation of clusters and the location of branch points during the synthesis of amylopectin clusters. They compared the amylopectin fine structure in internal lengths of B2 and B3 chains and cluster repeat distance among *be2b*, *be1a*, *be2b-be1a*, and WT. In addition, the previous study by Man et al. (2013) reported that inhibition of both BEI and BEIIb activities resulted in a significant increase in the size of amylopectin cluster in mature endosperm from a *japonica*-type rice cultivar. It is unclear how loss of both BEI and BEIIb affects the cluster size considering that the BEI’s role in amylopectin biosynthesis sharply contrasts from the BEIIb role (Figure 10; also see reviews by Nakamura, 2002, 2015, 2018). Anyhow, these discrepancies should be resolved in the further investigations.

## Conclusion

It is widely known that starch in cereal endosperm is the A-type crystalline polymorph whereas that in tubers is the B-type crystalline polymorph. Since BEIIb is specifically expressed in endosperm of cereals such as maize and rice, but not in tubers, this isozyme must play a critical role in the formation of the cereal-specific amylopectin cluster structure. In the present study, detailed analysis of chain-length distribution of amylopectin by the FACE method showed a marked decrease in short A and B1 chains in *ae* endosperm amylopectin. From these observations, we conclude that BEIIb was responsible for the formation of new short cluster chains and this resulted not only in the decrease in the average length of cluster chains but also in the increase in the number of cluster chains per a single cluster. These changes in the cluster structure of amylopectin induced by the *be2b* mutation caused the internal structure of starch granules from the A-type to B-type polymorph, as detected by XRD, SAXS, solid state ^13^C NMR, and SFG analyses. The SAXS analysis also showed that the size of amylopectin cluster was significantly shortened by loss of BEIIb activity in the endosperm when the starch granules were fully hydrated during analysis. The present results lead us to the new model of the amylopectin cluster structure, in which branch points are classified into two groups, as illustrated in Figure 10. We propose that branch points in the first group, which are mainly formed by BEI, are located in the basal region of the cluster constituting the amorphous lamellae. Since this role of BEI can be supplemented by other isozymes such as BEIIa and BEIIb at least to some extent (Nakamura, 2002; Satoh et al., 2003), the first branches are seen in rice endosperm having any BE-related genotypes; e.g., the *ae* mutant. In the second group branch points formed specifically by BEIIb are localized in the basal part of the crystalline lamellae and possibly also in the intermediate between the amorphous and crystalline lamellae, but not distributed throughout the cluster region. It is thought that this model can rationally explain the reason why the *ae* mutant starch not only exhibits the B-type polymorph in the endosperm, but also has high onset temperature of gelatinization and is highly resistant to attack by hydrolyzing enzymes (see review by Nakamura, 2018).

The present study also suggested the role of BEIIa in amylopectin biosynthesis in the culm where BEIIb activity was absent even in the WT rice, and BEIIa accounted for the major BE activity. It was striking to note that in the *ae* mutant amylopectin in the culm had more short chains of DP ≤ 14 and depleted intermediate and long chains compared with that in the endosperm, suggesting strongly that BEIIa was involved in the synthesis of short chains in the culm. In other word, BEIIa was considered to have the same role in amylopectin biosynthesis in the culm as BEIIb in the endosperm. We hypothesize that BEIIa was active in the culm whereas it was inactive in the endosperm in the presence of BEIIb. The transition between the active and inactive state of the BEIIa activity might be controlled by the capacity of BEIIb through its pivotal role in forming the protein-protein complex (see Crofts et al., 2017, 2018), although the mechanism remains to be resolved in the future study.

## Data Availability Statement

The raw data supporting the conclusions of this article will be made available by the authors, without undue reservation.

## Author Contributions

YN conceive and designed the study. YN and MO (biochemical experiments), TH and KK (XRD), KY and GMa (SAXS), and AMa, AMi, and GMi (solid state ^13^C NMR and SFG) conducted the research and analyzed the data. YN, TH, KK, GMa, and GMi wrote, read and approved the manuscript. YN and KK were responsible for the model of the amylopectin cluster structure in rice endosperm, shown in Figure 10. All the authors contributed to the article and approved the submitted version.

## Conflict of Interest

YN is employed by Starch Technologies Co., Ltd. The remaining authors declare that the research was conducted in the absence of any commercial or financial relationships that could be construed as a potential conflict of interest.
