# Supplementary figures and images for: Effects of BEIIb-Deficiency on the Cluster Structure of Amylopectin and the Internal Structure of Starch Granules in Endosperm and Culm of Japonica-Type Rice
Source: Front Plant Sci. 2020 Nov 17;11:571346. doi: 10.3389/fpls.2020.571346 (PMC7704622; doi:10.3389/fpls.2020.571346)

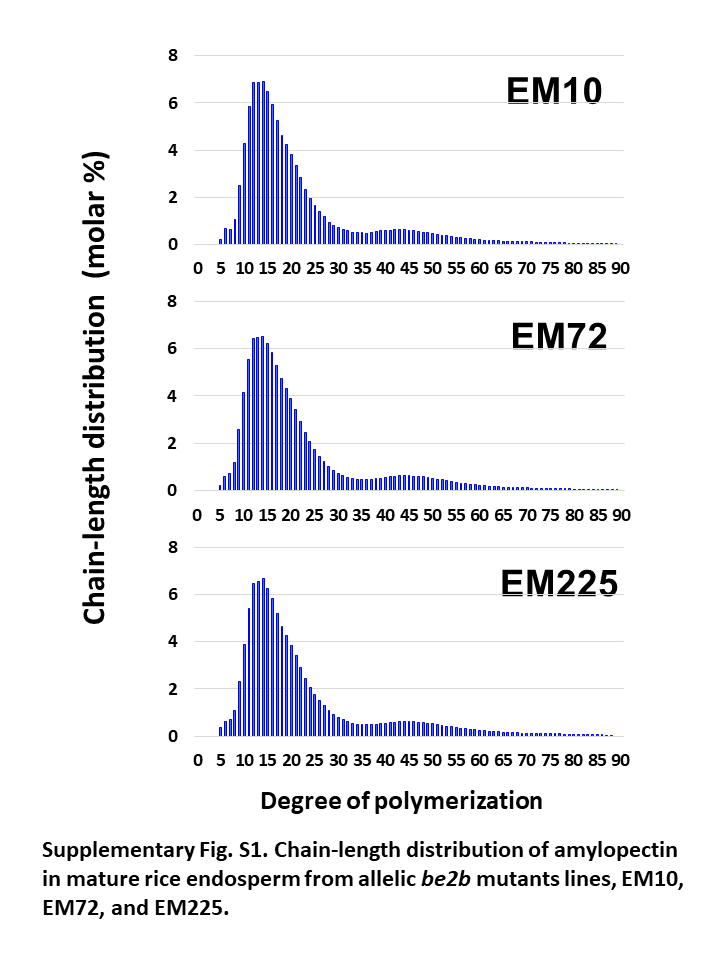

Supplement: Supplementary file 1 [file Image_1.TIF]

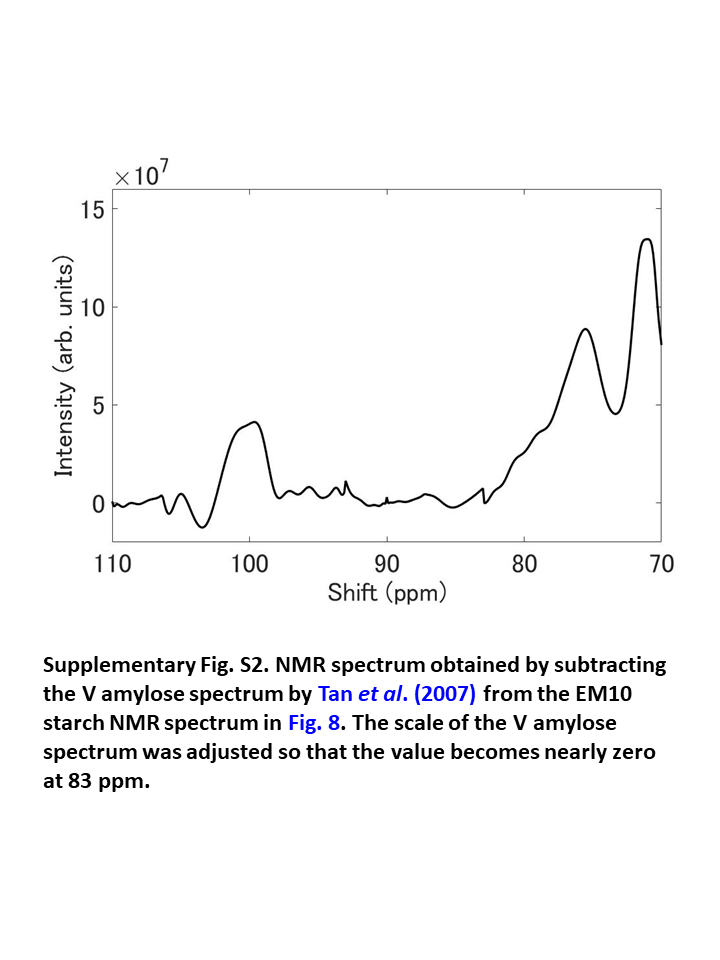

Supplement: Supplementary file 2 [file Image_2.TIF]
